# Stationary Phases for Green Liquid Chromatography

**DOI:** 10.3390/ma15020419

**Published:** 2022-01-06

**Authors:** Mikołaj Dembek, Szymon Bocian

**Affiliations:** Chair of Environmental Chemistry and Bioanalytics, Faculty of Chemistry, Nicolaus Copernicus University, Gagarin Str. 7, 87-100 Torun, Poland; m.dembek@doktorant.umk.pl

**Keywords:** pure water, liquid chromatography, chemically bonded stationary phases, polar-embedded stationary phases, subcritical water chromatography, SBWC, superheated water chromatography, SHWC, per aqueous liquid chromatography, PALC

## Abstract

Industrial research, including pharmaceutical research, is increasingly using liquid chromatography techniques. This involves the production of large quantities of hazardous and toxic organic waste. Therefore, it is essential at this point to focus interest on solutions proposed by so-called “green chemistry”. One such solution is the search for new methods or the use of new materials that will reduce waste. One of the most promising ideas is to perform chromatographic separation using pure water, without organic solvents, as a mobile phase. Such an approach requires novel stationary phases or specific chromatographic conditions, such as an elevated separation temperature. The following review paper aims to gather information on stationary phases used for separation under purely aqueous conditions at various temperatures.

## 1. Introduction

Liquid chromatography is a common separation technique that is commonly used in laboratories and various industries. Unfortunately, the liquid chromatographic separation process typically uses a significant number of organic solvents, and produces considerable quantities of harmful waste. This is a global problem that directly affects human life and the functioning of the environment. Thus, many ideas have been proposed to make chromatographic separation more environmentally friendly in the past decade. Solutions proposed by “green chemistry” are also increasingly used in high-performance liquid chromatography (HPLC). Replacing toxic solvents with “green” alternatives has attracted more and more interest, since it reduces the amount of waste products, which has a positive ecological effect, reduces costs, and improves the economic aspect. [1,2]. However, it must be emphasized that a purely aqueous mobile phase must have special requirements; thus, it is likely to increase costs in other areas, such as stationary phases or instrument operation at elevated temperatures.

To achieve utterly green chromatography, it is necessary to completely eliminate organic solvents and replace them with pure water [3,4,5,6,7] or supercritical carbon dioxide [8], while still being able to separate groups of compounds; this is only possible with the use of suitable phases. Currently, there are more and more references in the literature to commercially available or homemade stationary phases that allow working under such conditions. Knowledge of such phases is incredibly important for researchers involved in environmental and ecological fields in industries using chromatographic analysis.

This work collects information on the current state of knowledge of stationary phases used under purely aqueous conditions, describing the advantages as well as the requirements associated with the use of pure aqueous conditions; above all, it collects information about already-used, commercially available stationary phases working in 100% water in a structured and systematic manner.

**Figure 1:**
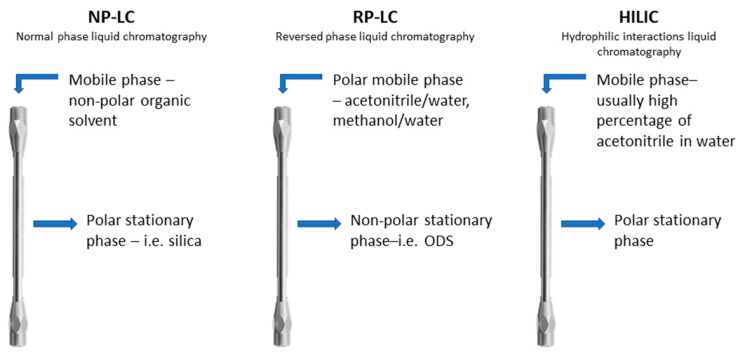
Characterization of the stationary phase and mobile phase in the NP-LC, RP-LC, and HILIC modes.

Separation techniques commonly applied in various laboratories and industries, including chromatographic techniques, consume large amounts of organic solvents. Acetonitrile, methanol, isopropanol, tetrahydrofuran, and additives (e.g., trifluoroacetic acid) are some of the toxic solvents commonly used in reversed-phase liquid chromatography (RP-LC). Considering “green” chromatography and thinking about our environment, these should be replaced with more environmentally friendly alternatives, e.g., water. A similar situation is observed in normal-phase liquid chromatography (NP-LC) and hydrophilic interactions liquid chromatography (HILIC), and the latter has gained more popularity in recent years. The general assumptions of these modes are shown in Figure 1.

Numerous “greener” ways to perform liquid chromatography were reviewed in our previous work [9]. The most common are downsizing the dimensions of the HPLC column [10]; the application of mobile phase additives, e.g., cyclodextrins [11] or surfactants; replacing organic solvents with water [3,4,5,6,7], ethanol [12], or supercritical CO_2_ [8]; and changes in the used stationary phases. The first solution involves the use of stationary phases modified with shorter carbon chains [13], materials based on core–shell particles [10], and the application of polar-embedded stationary phases [14]. Traditional stationary phases may also be operated at elevated temperatures [15]. Of those previously mentioned, this paper’s major topic is the application of stationary phases for HPLC enabling analysis in pure water. For such an approach, the proper selection of stationary phases is necessary.

A second approach to achieve more “green” analytical chemistry is to use mathematical data analysis methods. Using multivariate and multiway chemometric methods makes it possible to calculate separation conditions where the resolution will be increased, especially for more complex mixtures of analytes. Multiway methods in chemometrics are based on the analysis of higher order data than second-order data (matrix, second-order array)—for example, third-, fourth-, or higher order data. Such occurrence of data is common in chemistry, e.g., liquid chromatography coupled with mass spectrometry (LC–MS) [16,17]. This approach saves time, experimental work, consumption of organic solvents and analytes, and energy use [18,19,20]. Nevertheless, it will not always be a completely “green” solution, as organic solvents are used during the work, and must be treated as waste after the experiments; however, their consumption can be drastically reduced, so it is a more environmentally friendly alternative compared to commercially used methods.

A very “green” solution for liquid chromatography is to use software such as DryLab or ChromSword, among others [21,22]. These programs enable computer simulations of analyses, providing data on the optimal analysis conditions for a given mixture of analytes and chromatographic column. Thus, the analysis stage needed in order to optimize the separation method of a given group of compounds is omitted; hence, there is a significant reduction in organic solvents, energy costs, and working time.

When applying pure water as a mobile phase, there are two options for elution and separation: The first is to use specific stationary phases that increase the relative elution strength of water. The second option is to increase the temperature of the separation significantly. In both cases, the stationary phase properties are fundamental in the case of proper retention, elution, and thermal stability.

## 2. Purely Aqueous Conditions: Features and Requirements

As mentioned above, the use of pure water allows chromatographic analyses to be performed in a completely “green” manner. With industry and science growing faster and faster, the amount of waste generated is increasing. Therefore, we have no choice but to look for ways to ensure that modern and future-oriented analytical work does not add to environmental pollution. An extensive description of the problem and solutions was given by Welch et al. [23]. The past two decades have seen a growing interest in the application of pure water as a mobile phase. One can find many experimental works and several published review papers related to this issue [6,7,9,24,25,26,27,28,29,30,31]. Changes to standard liquid chromatography approaches are needed in order to make the separation in pure water possible. Two paths are possible: the first includes elevated temperatures, while the second remains at ambient temperatures but focuses on selecting appropriate stationary phases.

The magnitude of the temperature increase can vary between applications. However, the often-used phrase “high-temperature” is not exactly precise. Less precise terms such as “higher than room temperature” or “higher than 100 °C” and more precise terms such as “higher than the boiling point of the mobile phase solvent” or “in the range between 40 °C and 200 °C” appear in the literature [25,26]. Changes in water temperature affect a large spectrum of physicochemical parameters, which may dishearten analysts. Usually, only the influence on the viscosity is frequently discussed in the literature. Increases in eluent strength, increases in diffusivity, and changes in the dissociation rates of ionizable compounds are equally significant, but less frequently considered. Therefore, the overall benefits and difficulties associated with high-temperature analysis are not considered exhaustively [26]. Our previous paper described the discussion about temperature-dependent changes in water properties and their effects on analyses in pure water [9].

In RP-LC, increasing the temperature produces an effect analogous to that of increasing the organic solvent concentration in the mobile phase. The studies carried out by Bowermaster and McNair [32], and Chen and Horváth [33] show that a 1% increase in ACN concentration corresponds to a 5 °C increase in temperature. In contrast, an equivalent increase in methanol concentration corresponds to a 3.75 °C increase in temperature. Of course, the precise effect depends on the stationary phases used [34,35]. For example, in the methanol–water mobile phase, a temperature increase of 3.5 °C corresponds to a 1% increase in the concentration of the organic component, while a temperature increase of between 5 °C and 8 °C corresponds to a 1% increase in the concentration of acetonitrile in an acetonitrile–water mixture on polystyrene–divinylbenzene stationary phase (Hamilton PRP-1). Changing the stationary phase to Zorbax RX-C18 gives results of 1% of organic solvent concentration being equivalent to 2 °C for methanol–water and 3 °C for acetonitrile–water mobile phases [35]. The dielectric constant of water is reduced from 85 at 25 °C to 35 at 200 °C. Elevated temperatures make the water behave like an organic solvent; hence, it becomes a very effective solvent for separating weakly polar compounds [36]. Application of water at elevated temperatures allows experiments to be conducted under completely “green” conditions if the separated substances and the stationary phase are thermally stable [25].

**Figure 2:**
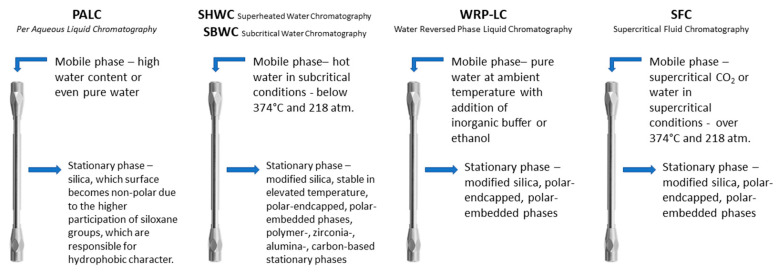
Characterization of stationary phases and conditions for using pure water as a mobile phase in different liquid chromatography modes.

If the properties of water depend on the temperature, changing the temperature makes it possible to separate substances with a full spectrum of polarity. High temperatures enable elution of non-polar compounds, moderate thermal conditions enable elution of weakly polar compounds, and low temperatures are sufficient for non-polar substances. Of course, this is limited by the thermal stability of the stationary phase. A higher temperature of the water changes the polarity and reduces the viscosity, but also reduces the analyte adsorption—which is exothermal—on the stationary phase. Depending on the conditions used and the authors, chromatography with water at elevated and ambient temperatures can be referred to by different terms. The modes with the description of the conditions of pure water as a mobile phase and features of stationary phases are shown in Figure 2.

More than 30 years ago, a study was conducted on separation in pure water [37]; sadly, it did not gain popularity. Satisfactory results were obtained when the surface of the stationary phase was modified with shorter hydrophobic alkyl chains not exceeding eight carbon atoms. Later studies confirmed that free silanols at the surface of silica particles play the main part in retention when such highly polar eluents as pure water are used; however, their excessive activity adversely affects the shape of the peaks and, therefore, the separation results. The use of even shorter carbon chains (up to C4) allowed us to obtain stable stationary phases with high coverage density. The problems associated with the attachment of longer alkyl chains are then avoided, since these phases have lower coverage densities and, at low contents of organic modifier in the mobile phase, tend to form chain bonds and decrease the stationary phase solvation [38,39].

The adverse effect of silanols on retention led to the search for new solutions, one of which is the use of polar-endcapped and polar-embedded stationary phases [40,41,42,43]. More details related to the structure, capabilities, and requirements of such phases will be described in the next section of this work.

An increase in water temperature causes changes in its eluotropic strength [44]. Unfortunately, the thermal stability of both column packing and analytes is a problem. In such a case, the next option is to change the mode of liquid chromatography. This issue can be overcome by using more polar stationary phases that allow for higher eluotropic strength of water.

Applying a purely aqueous mobile phase at room temperature is the most environmentally friendly mode of liquid chromatography, with advantages such as lack of toxic waste and low energy consumption. Usually, ambient conditions in liquid chromatography are interpreted as analyses performed below 60 °C. The literature suggests a new subcategory of this type of analysis—water-only reversed-phase liquid chromatography (WRP-LC)—and per aqueous liquid chromatography (PALC) is also among these techniques [45].

High elution strength of water is observed in hydrophilic interaction liquid chromatography (HILIC). Hydrophilic interaction chromatography is mainly used for the separation of polar compounds. The water content in HILIC is usually low, and it is impossible to carry out HILIC with pure water, due to the elution strength being too high.

An alternative to HILIC may be a silica hydride stationary phase applied in an aqueous normal-phase (ANP) system. ANP was named and described for the first time by Pesek et al. [46,47]. The changes in the retention of polar compounds from 0 to 100% water concentration present bimodal curves. The highest retention is observed at both ends—for high organic solvent content similarly to HILIC, and for pure water or high-water content [48,49,50].

The surface properties of silica gel in the purely water mobile phase are somewhat surprising. The hydrophilic character of silica is attributed to free silanol groups. Silanols, depending on the pH of the solution, may ionize. Bidlingmeyer et al. separated amines in HILIC on pure silica using pure water as a mobile phase [51]; the authors demonstrated that at high water content, the silica surface shows non-polar behavior. This is due to non-polarized siloxane groups that give the silica surface a hydrophobic nature [52]. Thus, the use of pure water makes the polar nature of the silica surface in HILIC change to non-polar. Therefore, A. dos Santos Pereira et al. proposed a new name for this reversed-HILIC mode—per aqueous liquid chromatography (PALC)—to distinguish this method from HILIC, RP-LC, and ANP-LC [53]. PALC requires the application of specific stationary phases.

The application of pure water as a mobile phase causes several problems. If the stationary phase is highly hydrophobic, such as C18, the bonded ligands may collapse to the support surface. This usually results in a decrease in retention, and reduces the reproducibility of the separations [3]. The second problem is that in RP-LC the elution strength of water may be too low to perform the elution. The opposite problem is observed in HILIC, where the elution strength is too high and prevents retention.

When using water at elevated temperatures, further constraints arise. Although the elution strength, viscosity, surface tension, and many other properties of water are favorably altered, high temperature places some limitations. A stationary phase operating under these conditions must be thermally stable. The substances to be analyzed must also exhibit thermal stability over the temperature range used. Degradation of all or some of the analytes of the tested mixture will completely disturb the results obtained. The thermal instability of analytes can be circumvented in several ways. The lower viscosity of the purely aqueous mobile phase causes low backpressure, enabling the use of much higher flow rates for SBWC separation; as a result, the degradation of analytes is reduced, because the analysis time and, consequently, their exposure to high temperatures, is reduced. Thus, analyte degradation is no longer a significant problem when using the SBWC technique for low-molecular-weight compounds [6].

The use of elevated temperatures is also associated with the use of appropriate instrumentation. It is necessary to ensure a stable temperature throughout the analysis, pre-heating of the mobile phase, and a suitable thermostat for the column [6,54].

## 3. Stationary Phases Used in Pure Water Conditions

The first reported separation using subcritical water as a mobile phase was carried out by Smith and Burgess in 1996 [55]. The separations of several phenols, parabens, and barbiturates on a polystyrene–divinylbenzene (PS–DVB) stationary phase were carried out at 210 °C. The authors demonstrated that retention factors of separated substances decrease with increasing temperature. Compared to conventional reversed-phase conditions, superheated water separation at 180 °C provides a similar result to a 20:80 acetonitrile–water mobile phase at ambient temperature. No degradation or oxidation of any analytes or the stationary phase structure was observed in the experiments performed. Thus, the study concluded that SHWC, compared to the conventional RP-LC technique, gives better separation and allows shorter analysis times [55].

As discussed above, the thermal stability of analytes can be an obstacle to using water at elevated temperatures. The thermal stability of analytes was investigated by Thompson and Carr [7,56] in the case of drugs and alkaloids; they concluded that in order to not affect the analytes’ stability, it is necessary to reduce the analysis time. Doing so will allow even compounds that degrade at high temperatures to be separated, as the short residence time in the elevated-temperature column will not affect their stability [6]. The observed rule also has some exceptions; several other cases have been reported in which degradation has occurred. Thiamine can only be analyzed below 50 °C, while a temperature of 160 °C results in many degradation products [57]. Nitrobenzene was degraded above 220 °C using a PS–DVB column; however, a reduced temperature and a less retentive phase allowed the decomposition to be prevented [7]. Even polycyclic aromatic hydrocarbons can degrade; analyses in the range of 100–350 °C and an extensive range of heating times from 10 to 240 min in pressurized hot water showed that even at the shortest time interval, they degraded above 300 °C; at longer times, damage to the structure of the compounds occurred even at 100 °C [58].

Despite the examples of degradation described above, SHWC can work for substances with a broad polarity range, mainly due to decreased water viscosity and polarity with increasing temperature. Several commercial chromatographic columns may be used to separate low-molecular-weight compounds using supercritical water. By selecting a suitable stationary phase or suitable analysis conditions, working with pure water at room temperature is also possible. These materials are described in the literature and listed in Table 1, Table 2, Table 3 and Table 4.

### 3.1. Instability of Stationary Phases—A Motivation to Search for Solutions

In most cases, the thermal stability of analytes is not a significant problem, and a wide range of substances may be analyzed. Another serious problem is the thermal stability of the stationary phase used. Most of the materials used as stationary phases that are stable under ambient temperature HPLC may not be stable under superheated water conditions. Despite the temperature range, the van ’t Hoff relationship remains linear for both ambient temperature HPLC and SHWC [59,60].

One of the leading examples of a thermally unstable stationary phase is the most commonly used chromatographic material—octadecyl-modified silica (ODS)—which undergoes degradation at temperatures above 80 °C [7]. For this reason, it is essential to develop new materials or modify existing ones in order to ensure their thermal stability when using pure water as the only eluent. When analyses are carried out with ODS silica materials at water contents higher than 95%, a sudden decrease in retention times occurs while maintaining efficiency. This is due to a phenomenon called the “phase collapse” effect, which is associated with the stacking of long-chain alkyl ligands [61]. In this case, the adsorptive surface area of the stationary phase is strongly reduced. However, the “phase collapse” process is reversible, and this can be achieved by using a mobile phase with a high organic modifier content. This effect is explained by the de-wetting of the phase surface, which lowers the effective volume of the column by removing water molecules from the pores [62].

Many attempts have been made to prevent this effect. For example, polar groups were incorporated between the hydrophobic octadecyl chains and the silica surface; this led to the obtaining of mixed hydrophilic–hydrophobic phases. Such materials have improved wettability in high-water-content or even in purely aqueous mobile phases. Novel materials were named polar-endcapped, polar-embedded, or aqueous stationary phases [39,63].

With SHWC, and using phases with embedded polar groups, changes in retention were also observed. This was achieved by cooling the column and leaving it without flow for a period of time; however, this effect can occur suddenly—even between performed analyses. The overall behavior of stationary phases under aqueous conditions was described in detail by Walter et al. [64].

Consequently, the inability to successfully use commercial octadecyl phases has forced the search for other stationary phases that are thermally stable under pure water conditions. Currently, several chromatographic phases are used in SHWC and other techniques using pure water as the only eluent. The most common are: modified silica phases, polymer phases, zirconia-based phases, other metal oxide phases, carbon phases, and hybrid phases [6,7].

#### 3.1.1. Silica-Based Packing Materials

Despite the problems mentioned above, conventional silica stationary phases are applied in water conditions. Unfortunately, the temperature range in which they can be operated is low; additionally, in most cases, their lifetime is reduced. This is mainly observed in the case of ODS materials.

Problems that arise during analyses at elevated temperatures mainly focus on the degradation of analytes. The occurrence of such a phenomenon is not necessarily due to the use of pure water or elevated temperatures. For example, using a Zorbax RRHD Eclipse Plus column for the separation of coumarin, vanillin, and ethyl vanillin gave ethyl vanillin as a degradation product. However, the use of other columns under the same conditions did not cause degradation of the analyte [65]. This confirms that the degradation resulted from analyte–stationary phase interactions, rather than from factors associated with the application of pure water or elevated temperature. In addition, it can be concluded that by having a wide range of stationary phases commercially available, the reasons for degradation can be checked efficiently and avoided.

The thermal stability of the column can be measured—simply perform a mixture separation analysis and then repeat the analysis after a specified volume has passed through the column. An example study was performed by He and Yang [66], investigating the change in retention of a mixture of caffeine, benzene, and methyl benzoate. Nucleosil C18 AB phase degradation occurred when passing more than 8000 column volumes at 100 °C. The reason for increasing the retention of the polar compound and decreasing the retention of the non-polar compound is the gradual removal of the C18 chains from the silica surface, causing a decrease in its non-polar character.

Studies also confirm that stationary phases operating over a wide pH range have greater thermal stability [67]. In addition, those based on ethylene-bridged hybrid (BEH) technology are currently the most thermally stable and pH-stable silica-based columns [68].

Using elevated temperatures can be problematic, so performing analyses at room temperature using pure water is highly desirable. The PALC technique mentioned above allows operation under highly aqueous or fully aqueous conditions while maintaining ambient temperature; it has become competitive with HILIC and, combined with the environmentally beneficial aspect, interest around it has increased. Initially, in the literature, one may encounter the term reversed HILIC; however, this suggests that the retention mechanism occurs according to RP-LC, for which reason the term PALC has been introduced.

The application of Zorbax-Rx-SIL to separate catecholamines in standard HILIC was unsuccessful on superficially porous particles [69]; however, it was successful using PALC, obtaining excellent peak shapes and high efficiency. In a later work, Gritti and dos Santos Pereira explained the retention mechanism occurring in PALC, and described the effect of the water-rich mobile phase on the separation efficiency [70,71]. The adsorption mechanism was explained by determining adsorption isotherms using frontal analysis (FA) on pure porous silica (HALO) core–shell structures; this enabled determination of the differences in mechanisms between HILIC and PALC. The low acetonitrile content in PALC makes the silica surface strongly heterogeneous; in contrast, in HILIC, the high acetonitrile content makes the free silanols responsible for the retention: single silanol groups and geminal and/or vicinal hydroxyl groups [70]. HALO column efficiency measurements showed a lower value of height equivalent to a theoretical plate (HETP) for PALC compared to HILIC [71].

PALC gained wider popularity in the following years, and various materials were tested as a stationary phase. At first, Li et al. used a polysaccharide-modified stationary phase (PMSP) to separate six polar substances: melamine, vitamin B2, vitamin B6, caffeine, benzoic acid, and hydroquinone [72]. Subsequent detailed studies proved that PALC could obtain retention factors as good as HILIC for separating polar compounds. Modifying silica particles with the functionalized carbon nanoparticles (CNPs) obtained from corn stalk soot allows polar stationary phases to be obtained. Such materials can work with both water-rich and acetonitrile-rich mobile phases [73].

PALC also enables the use of hybrid silica materials such as a 1.7 µm ethylene-bridged hybrid silica stationary phase (BEH HILIC); its application to the separation of 12 imidazole-based ionic liquids’ cations showed that the PALC system could enable retention with both hydrophobic and ion-exchange mechanisms [74]. Another application of PALC was used by Matos et al., combining it with size-exclusion chromatography (SEC) to determine the water-soluble organic matter (WSOM) content in atmospheric aerosols collected from urban areas during different seasons [75].

Due to its positive ecological and economic aspects, PALC is sometimes used as a complementary technique to RP-LC. Detection of four types of biogenic amines and five nucleic bases and nucleotides was performed on a silica-based column with Congo red molecules attached (Sil-CR) using PALC [76]. The results obtained showed better separation with PALC than using HILIC, which consumes large amounts of organic modifiers.

In the RP-LC technique, applying pure water as a mobile phase requires surface modification of the silica-based stationary phase. These phases have embedded polar groups to provide a mixed retention mechanism and allow water to interact with the silica surface. The polar-embedded and polar-endcapped phases will be discussed in subsequent sections of this paper. Commercially available silica-based stationary phases and the groups of compounds separated on them are summarized in Table 1.

**Table 1:** Literature dataset of silica-based chromatographic columns and groups of chemical compounds separated by them using pure water.

Chromatographic Column	Group of Chemical Compounds																						
	Alcohols	Aliphatic and Aromatic Ketones	Alkyl Benzenes/Chlorinated Benzenes/Benzene Derivatives	Anilines	Aromatic Acids	Aromatic Hydrocarbons	Barbiturates	Benzoates	Caffeine Derivatives	Carbohydrates	Chlorophenols	Diethyl Phthalates	Model Drugs	Nucleobases	Parabens	Phenols	Polychlorinated Biphenyls (PCBs)	Polycyclic Aromatic Hydrocarbons (PAHs)	Polyhydroxybenzenes	Pyridines	Steroids	Water-Soluble Vitamins	Literature
Akzo Nobel Kromasil Eternity-2.5-C18								x		x		x		x	x			x					[68]
Chromatorex C-18														x		x			x	x			[35]
Daiso (ODS-BP)										x													[77]
Develosil C30-UG-5	x																						[78]
Fuji Silysia Chromatorex 10 μm											x												[79]
Hyperprep C18			x																				[80]
Hypersil BDS C18									x				x										[66,81,82]
Interchim Uptisphere Strategy C18-2 and C18-3								x		x		x		x	x			x					[68]
Kromasil-C18			x						x														[83]
L-column ODS Chemicals Evaluations and Research Institute, Japan															x								[84]
Nanoporous glass modified with TFPS and ethyl acetate			x																				[80]
Novapak C18													x			x						x	[57,85]
Nucleodur-C18			x																				[83]
Nucleosil C18 AB			x	x					x							x							[66,86]
ODS Chrompack	x																						[87]
Partisil ODS2 ES			x													x		x					[88]
Silica-silicon-based ethyl-bridged hybrid C18 5 µm	x		x																				[89]
Spherisorb octadecylsilane (ODS)-bonded silica						x	x								x								[90,91]
Spherosil XOA (200, 600, 800 mesh)-C18	x					x										x							[92]
Supelco Ascentis Express C18 (fused-core)								x		x		x		x	x			x					[68]
Xselect CSH C18								x		x		x		x	x			x					[68]
Xbridge Amide								x		x		x		x	x			x					[68]
Xbridge C18		x			x								x										[81,93,94]
XTerra MS C18 and Xterra phenyl organic/inorganic hybride		x	x						x														[83,95]
Xterra RP C8		x			x								x								x		[93,96,97]
Xterra RP C18		x	x		x				x												x		[83,93,97]
YMC Triart C18								x		x		x		x	x			x					[68]
Zorbax RX-C-18	x													x		x			x	x			[35]
Zorbax RX-C-8									x							x							[66,82]
Zorbax-ODS																	x	x					[98]

#### 3.1.2. Polymer-Based Stationary Phases

Polymeric materials usually exhibit higher thermal stability. Such fillings are successfully used in elevated-temperature chromatography, e.g., for size-exclusion chromatography; thus, it is reasonable to expect them to be the most thermally stable chromatographic materials for analyses using superheated water as a mobile phase. Among polymer materials, two of them are most commonly used in SHWC: polystyrene divinylbenzene PLRP-S (PS–DVB) [55,57,81,83,90,99,100,101], and crosslinked polymer PRP-1 by Hamilton [35,36,77,87,89,102,103]. All polymeric stationary phases and the groups of compounds separated on them are summarized in Table 2. The thermal stability of these phases is between 100 °C and 200 °C. Polymer-based phases are more thermally stable than silica-based phases; they are therefore used in conditions of prolonged operation at temperatures higher than 200 °C. However, when comparing retention times and temperature, using the silica phase is sometimes more advantageous, because the analysis time is shorter and does not require such high temperatures. Of course, the limiting parameter for using these phases is their lifetime, so for long-term use, polymeric phases are mostly used [90]. The literature also mentions new polymeric phases that contain attached amino acids that allow them to operate in heated water up to 150 °C for 500 h [104]. Despite better thermal stability, polymeric stationary phases also have some disadvantages compared to silica-based materials, e.g., lower column efficiency; this is likely related to the high retention capacity; thus, groups of moderately polar compounds such as alkyl- and aryl ketones can be eluted only at elevated temperatures.

**Table 2:** Polymer-based stationary phases used to separate groups of compounds in pure water.

Chromatographic Column	Group of Chemical Compounds																									
	Alcohols	Aldehydes	Aliphatic and Aromatic Ketones	Alkyl Benzenes/Chlorinated Benzenes/Benzene Derivatives	Amino Acids	Anilines	Aromatic Acids	Barbiturates	Benzoates	Caffeine Derivatives	Carbohydrates	Carboxylic Acids	Chlorophenols	Diethyl Phthalates	Model Drugs	Nucleobases	Parabens	Phenols	Polycyclic Aromatic Hydrocarbons (PAHs)	Polyethylene Glycols	Polyhydroxybenzenes	PTH–Amino Acids	Pyridines	Sulfonamides	Water-Soluble Vitamins	Literature
Aminex HPX 87-strong cationic resin	x										x															[105]
Oasis polymer															x											[81]
P(NIPAAm-co-BMA-co-DMAPAAm) modified silica																						x				[106]
P(NIPAAm-co-tBAAm-co-AAc) modified silica																						x				[106]
PL HiPlex 8µm H			x				x																			[93]
PLRP-S PS–DVB Polymer Laboratories			x	x			x	x		x					x		x	x						x	x	[56,60,63,81,83,85,90,93,99,100,107,108]
Poly(GMA-co-EDMA) particles						x												x					x			[109]
Polystyrene-Coated Zirconia (PS-ZrO_2_)																		x								[54]
PRP-1 Hamilton	x	x			x	x				x	x	x	x			x		x		x	x		x			[35,66,77,79,82,86,87,102,110,111]
Showa Denko Shodex ET-RP1 4D									x		x			x		x	x		x							[68]

#### 3.1.3. Zirconia-Based Stationary Phases

Silica, due to the low pH of the point of zero charge (pH_pzc_), allows only cation exchange under neutral pH conditions. The dissociation of silanols then occurs, and the silica surface acquires a negative charge. Therefore, chromatography of basic compounds on silica is troublesome, because it requires a significant change in pH—which adversely affects the attached ligands or the silica itself—or the use of ion-exchange displacers or anionic ion-pairing agents [112,113,114].

Metal oxides—especially zirconium, aluminum, and titanium oxides—have much higher pH_pzc_, which means they do not have a negative surface charge, so no electrostatic interactions will occur; they behave as amphoteric ion exchangers, so anionic or cationic exchange can occur depending on the pH. Due to the presence of Lewis acid sites, the additional ligand exchangeability works to the advantage of using these supports [112,113].

Carr et al. were the first research group to examine the use of zirconium oxide as a potential packing material for chromatography columns [113,115]. This raised issues about modifying its surface with alkyl ligands. The modification required special conditions and additives. Finally, it turned out that among metal oxides, only zirconia could be used as effective chromatographic material.

Zirconium oxide has many adsorption sites, so it can be easily modified. This action favors a wide adaptation of the use of zirconium-based stationary phases. In general, there are three types of zirconium oxide modification: (1) dynamic, where the mobile phase contains a strongly interacting Lewis base; (2) permanent, where the zirconium oxide surface is permanently silylated as a result of binding to adsorption sites; and (3) physical screening, e.g., coating the zirconium surface with polymer or carbon. The most commonly used approaches are zirconia encapsulated by polybutadiene (PDB)—which is also known as a ZirChrom-PDB [81,83,106,116,117,118,119,120,121,122]—and zirconia encapsulated by polystyrene (PS), called ZirChrom-PS [54,66,120]. Other zirconia-based packings include carbon-coated zirconia (CARB)—ZirChrom-CARB [81,117,123]—or a secondary bonded C_18_ zirconia-based stationary phase (DiamondBond) e.g., ZirChrom-DB-C18 [83]. All zirconium- and aluminum-based stationary phases are listed in Table 3.

Zirconium oxide used as a support in chromatographic analyses has good thermal stability and is stable under pure water conditions. For ZirChrom-PDB, the manufacturer recommends an upper temperature limit of 150 °C; however, the thermal stability of this material has been observed up to 200 °C. ZirChrom-PDB was tested by Wu et al. [116], who showed that the upper limit of the column’s thermal stability reaches 260 °C. The ZirChrom-PDB and ZirChrom-CARB columns tested by Kephart et al. [117] reached thermal stability of 370 °C and 300 °C, respectively, using a pressure of 75.8 MPa. Compounds from the phenol and alkylbenzene groups were successfully separated on both stationary phases.

It is also important to note that complete removal of CO_2_ from the mobile phase is necessary before working in pure water, as it binds to the Lewis acids sites, completely changing the surface character of the stationary phase. Therefore, it is necessary to boil the water before use as a mobile phase, degas it, or use a scrubber or pre-column [113].

**Table 3:** Collection of zirconium- and aluminum-based stationary phases used to separate the listed groups of compounds in pure water.

Chromatographic Column	Group of Chemical Compounds									
	Alcohols	Alkyl benzenes/Chlorinated Benzenes/Benzene Derivatives	Caffeine Derivatives	Diethyl Phthalates	Flavones	Model Drugs	Phenols	Steroids	Triazole Fungicides	Literature
Alumina Keystone Scientific		x					x			[86]
PBD-encapsulated zirconia	x									[116]
ZirChrom–Carb		x				x				[81,117]
ZirChrom-DB-C18		x	x							[83]
ZirChrom-PBD	x	x	x	x	x	x	x	x	x	[81,82,83,106,118,119,124]
ZirChrom-PS			x		x					[66,120]

#### 3.1.4. Carbon-Based Packing Materials

Of the carbonaceous materials, only porous graphitized carbon (PGC) has found application in liquid chromatography. This material is obtained by immersing silica grains in a phenol–formaldehyde mixture. Next, gradual heating results in the formation of a phenol–formaldehyde resin, which is then carbonized at 900 °C in a nitrogen atmosphere. Then, the silica template is removed using KOH. The final carbonaceous material is heated at 2500 °C in an oxygen–argon atmosphere. The resulting material consists of uniform porous carbon, with a known pore structure and a surface oxidized under oxygen conditions.

Some chromatographically relevant features characterize carbon support—it is highly thermally resistant, with a well-reproducible structure and a surface that does not show the presence of charge during chromatographic operation. The specific surface of this material is suitable to provide retention, and to maintain linear capacity over a wide range of analyte concentrations. The porous structure does not exhibit the presence of micropores, and the pore size is larger than 10 nm, which ensures efficient mass transfer to and from the solution from the particles. PGC is resistant to highly interfering solvents; thus, no swelling, shrinkage, or dissolution occurs. This material is stable over the entire pH range and at high salt concentrations; it exhibits a unique retention mechanism and selectivity. Unlike common ODS phases, it has stereoselective properties that allow it to separate isomers and compounds with very similar structures [125].

The first PGC-packed columns were made by Hypersil—a division of Thermoquest Corporation; the columns are now sold under the Hypercarb name. In 1991, two Japanese groups—one from Tonen Corporation and the other from Nippon Carbon Company and Tosoh Corporation—released porous graphites obtained via a completely different procedure. Despite the differences in how these materials are obtained, their chromatographic properties do not differ. The type of chromatographic columns filled with PGC depends on the preparation procedure and the silica used for templating. Thus, several versions of such columns are commercially available, although their chromatographic properties coincide [125].

A more detailed study of Hypercarb shows that it is a very high-thermal-strength column. The dependence of *log k* on 1/*T* for this column, in the temperature range 20–180 °C, is linear, with a very good fit, which proves the independence of ∆H and ∆S from temperature over a wide range, regardless of the mobile phase composition—even in pure water. Although zirconium-based stationary phases exhibit nonlinear Van ’t Hoff plot relationships, for all of the substances studied by Guillarme, the Hypercarb relationships remained linear, indicating no effect of temperature on the eventual surface modification of the carbon material and excellent regularity and rigidity [83].

The disadvantage of PCG is the contaminants that form due to the high activity of the carbon materials, resulting in a problem with the formation of asymmetric peak shapes in the chromatograms. The second disadvantage of PGC columns is that their efficiency declines with time. Although carbon materials have high thermal stability, the significant difference in thermal expansion compared to a stainless steel column causes high mechanical stresses, resulting in a loss of separation properties of the bed [126]. All carbon-based stationary phases are listed in Table 4.

**Table 4:** Overview of carbon-based stationary phases and groups of compounds that were separated using pure water as a mobile phase.

Chromatographic Column	Group of Chemical Compounds														
	Alcohols	Aldehydes	Aliphatic and Aromatic Ketones	Alkyl Benzenes/Chlorinated Benzenes/Benzene Derivatives	Amino Acids	Caffeine Derivatives	Carbohydrates	Carboxylic Acids	Model Drugs	Nucleobases	Peptides	Steroids	Triazine Herbicides	Morphine-Based Opiates	Literature
Hypercarb 5 µm	x			x	x	x	x	x					x		[83,87,102,124,126]
Hypercarb PH porous graphitic carbon									x						[81]
Porous graphitized carbon (PGC)		x	x		x						x	x		x	[125]

## 4. Stationary Phases with Integrated Polar Groups

Surface modifications of silica by placing both polar and non-polar parts on the phase surfaces appeared in the early 1990s. This was due to the search for materials that allow for effective and selective operation in a wide range of organic modifier concentrations. This phase structure allows a mixed retention mechanism, among other things. Both polar and non-polar compounds are retained through interactions with hydrophobic and hydrophilic parts of the stationary phase [41,42,43].

**Figure 3:**
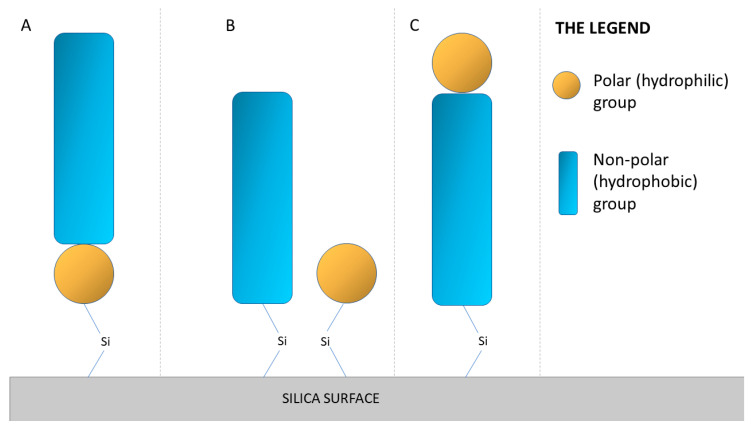
Schematic representation of the possibility of incorporating a polar group into the structure of a non-polar phase. (**A**): polar–embedded; (**B**): polar–endcapped; (**C**): polar–headed [40].

Among the stationary phases possessing a polar group and a hydrophobic ligand, we can distinguish three types: Polar-embedded stationary phases are obtained by attaching a polar group to the silica surface in place of a silanol, and then a hydrophobic part is attached to it; this construction of the phase with an incorporated polar group and an attached non-polar component allows for better water solvation at high-water mobile phase conditions. Polar-endcapped stationary phases are obtained in a two-step process. First, the surface of the stationary phase is modified by the attachment of non-polar parts, such as alkyl chains; in the second step, polar groups are endcapped by a specific reagent that possesses a polar group. The methodology is analogous to typical hydrophobic endcapping. Polar-headed stationary phases have a non-polar part attached to the silica surface, and the hydrophilic group is located at the end [40]. A schematic representation of these phases is shown in Figure 3.

Such materials have a series of advantages; they can be used in an RP-LC system and, compared to typical alkyl-bonded stationary phases, they provide a different selectivity for polar compounds. Polar-embedded stationary phases also allow operation using highly polar mobile phases, so they can successfully be used for separations using 100% water. Polar groups reduce the influence of residual silanols that result in the absence of tailing of basic chemical compound peaks [14,39,127,128,129,130,131,132,133,134,135,136,137,138].

Many papers have now been published demonstrating the separation of substances with a wide range of polarities using pure water at room temperature. Successful separations of alkylanilines using an alkylamide chemically bonded phase were described in 1994 by Buszewski et al. [139]. Some attempts have been made to apply octadecyl stationary phases modified with strongly positively/negatively charged surfactants; such stationary phases were used for the separation of nucleosides and nucleic bases [140,141,142]. Compounds of different hydrophobicity were separated using poly(N-isopropylacrylamide)-modified silica [143]; it was shown that it is possible to separate steroids efficiently at ambient temperature; the authors also investigated the influence of the temperature on the resolution. It must be remembered that in a single-component mobile phase (pure water), the separation temperature is the only parameter that can influence the retention and separation; thus, the proper choice of the stationary phase for a given mixture to be separated is essential. Another polymeric material used in pure water separation was a polyethylene glycol stationary phase (Supelco Discovery HS PEG), which was used by Šatínský et al. for the separation of analytes of different polarities [3]. Kiridena et al. applied a polar-endcapped chromatographic column (Synergi^TM^ Hydro-RP) at room temperature and elevated temperatures, but below 65 °C [63]. Recent work indicates the possibility of using polar-embedded phases in both RP-LC and HILIC systems to separate polar compounds [14].

**Figure 4:**
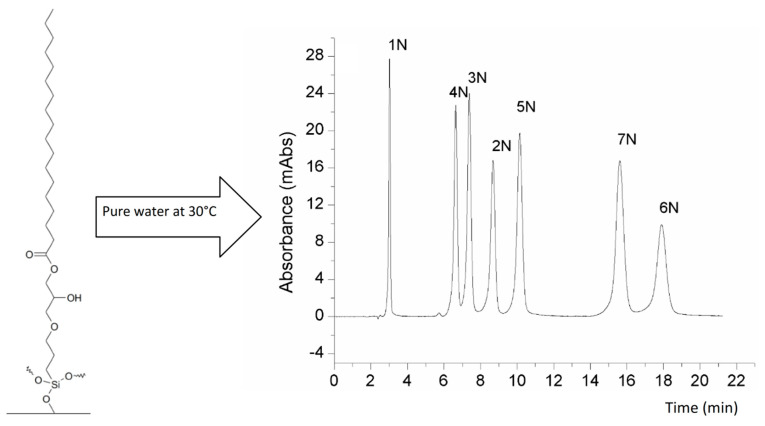
Separation of seven nucleosides on a polar-embedded stationary phase (Ester-C18) using pure water at low temperatures as a mobile phase. Description of the compounds—1N: uridine; 2N: guanosine; 3N: 1-methylinosine; 4N: thymidine; 5N: 1-methylguanosine; 6N:N2-methyloguanosine; 7N: adenosine. Adapted from ref. [14].

The ability to freely select the polar group results in stationary phases giving an efficient performance at room temperature of the mobile phase, with reasonable retention times. The polar-embedded stationary phase to be applied in the pure water mobile phase has to meet three basic requirements: First, it must provide retention of analytes, and this should be selective for different substances. Second, pure water must elute analytes in a reasonable time. Finally, it must provide unique selectivity and specific surface properties due to the presence of a polar group and a hydrophobic ligand [14,127]. The successful separation of a series of nucleic bases, nucleosides, and purine alkaloids is described in a recent paper by Bocian and Krzemińska. Pure water was the only mobile phase component at 30 °C, and a standard HPLC system was used for the analyses. As a stationary phase, the N,O-dialkylphosphoramidate phase [131] and a series of ester-bonded phases [127] were used. The obtained results confirm that applying polar-embedded stationary phases may enable water-only separation at ambient temperature conditions [14]. Exemplary structures of stationary phases used for purely aqueous separation are presented in Figure 4.

Another advantage of such stationary phases is that they may also be operated in RP-LC and HILIC systems. Nevertheless, the separation using pure water at room temperature as the only eluent is the most exciting and most “green” application. Unfortunately, in such a case, the selectivity of the separation results only from the stationary phase. However, there is an option to modify the temperature. Thus, designing and synthesizing new stationary phases with a different selection of polar and non-polar parts will certainly allow different selectivity, and will also lead to the optimization of techniques using pure water in liquid chromatography. These are options that can be implemented in the low-temperature range; broader possibilities appear when the water temperature is raised significantly.

## 5. Summary

The separation in liquid chromatography can be performed using pure water as a mobile phase. The application of pure water is environmentally friendly, and it is the best option for “green” chromatography.

Separations in purely aqueous conditions may be carried out at room temperature using specific polar-embedded stationary phases; however, in such a case, the selectivity depends almost entirely on the stationary phase. The second option is separation at elevated temperatures—superheated water chromatography. The change in water temperature changes its polarity, dielectric constant, viscosity, surface tension, and many other properties; as a result, the elution strength of water increases. Unfortunately, another problem may arise in this case—the thermal stability of both separated substances and stationary phases. Thus, several stationary phases based on silica gel, carbon, polymers, or metal oxides were obtained for application in purely aqueous separation. More new stationary phases will likely be developed soon, and “green” liquid chromatography will gain popularity in the coming years.

For completely “green” chromatography, it is necessary to use only nontoxic, environmentally friendly solvents; such solutions include carbon dioxide, ethanol, and water. In this review paper, we focus on water; its use as the sole component of the mobile phase presents many challenges to chromatographers, including viscosity, high elution force, dielectric constant, degradation of stationary phases, hydrolysis, and decomposition of analytes. Thus, it is necessary to select either an appropriate analysis method—as we discussed in our previous work [9]—or an appropriate choice of stationary phase, as described in this work; often, the two can be combined. The most convenient option is to use a stationary phase that allows the use of water without increasing the temperature or using additives in the form of salts or buffers; such solutions include phases with silica, polymer, zirconium or aluminum oxide, or carbon supports. In recent years, silica-based phases with embedded polar groups have proven to be very promising; they are thermally stable and, through the mixed hydrophilic–hydrophobic nature of the surface, allow operation under pure water conditions without the use of additives or elevated temperatures. The goal is to find phases that allow us to simultaneously understand retention mechanisms, control selectivity (e.g., by selecting appropriate groups at the functionalization stage of the stationary phase), and obtain good resolution and efficiency. The phases mentioned above were found by attempting to change the support; in contrast, polar-embedded and polar-endcapped stationary phases open possibilities to manipulate the parameters via a good selection of polar and non-polar groups attached to the silica surface at the synthesis stage; this provides an extensive range of possibilities for the preparation of different phases. In our opinion, it is on these phases that research aimed at finding stationary phases to work under pure water conditions should focus.
